# The pathogens of secondary infection in septic patients share a similar genotype to those that predominate in the gut

**DOI:** 10.1186/s13054-022-03943-z

**Published:** 2022-03-24

**Authors:** Sucheng Mu, Hao Xiang, Yuezhu Wang, Wei Wei, Xiangyu Long, Yi Han, Zhongshu Kuang, Yilin Yang, Feixiang Xu, Mingming Xue, Zhimin Dong, Chaoyang Tong, Huajun Zheng, Zhenju Song

**Affiliations:** 1 Department of Emergency Medicine, Zhongshan Hospital, Fudan University, 180 Fenglin Road, Shanghai, 200032 China; 2 NHC Key Lab of Reproduction Regulation (Shanghai Institute for Biomedical and Pharmaceutical Technologies), Fudan University, 2140 Xietu Road, Shanghai, China; 3 Shanghai-MOST Key Laboratory of Health and Disease Genomics, Chinese National Human Genome Center at Shanghai and Shanghai Institute for Biomedical and Pharmaceutical Technologies, 250 Bibo Road, Shanghai, China; 4 Shanghai Key Laboratory of Lung Inflammation and Injury, 180 Fenglin Road, Shanghai, China; 5 Shanghai Institute of Infectious Disease and Biosecurity, School of Public Health, Fudan University, 138 Yixueyuan Road, Shanghai, China

**Keywords:** Sepsis, Gut microbiota, Bacterial translocation, Secondary nosocomial infection

## Abstract

**Background:**

Secondary nosocomial infections, which are commonly caused by *carbapenem-resistant Klebsiella pneumoniae* (CRKP) and *vancomycin-resistant Enterococcus faecium* (VRE), often develop in septic patients. This study aimed to identify the origin of secondary systemic pathogens and reveal the underlying mechanism of infection.

**Methods:**

In this prospective, observational case–control study, a total of 34 septic patients, 33 non-septic intensive care unit (ICU) patients and 10 healthy individuals serving as controls were enrolled. Three hundred and twelve fecal samples were collected and subjected to 16S rRNA gene amplicon sequencing. Metagenome sequencing was performed to identify the homology between dominant CRKP or VRE in the intestine and pathogens isolated from secondary infectious sites. C57/BL mice were established as pseudo germ-free animal model by pretreatment with broad-spectrum antibiotics for two weeks.

**Results:**

The abundance and diversity of the gut microbiota in septic patients was drastically decreased one week after ICU admission, potentially leading to the enrichment of antibiotic-resistant bacteria, such as CRKP. Furthermore, secondary bloodstream and abdominal infections caused by CRKP or VRE in septic patients occurred after intestinal colonization with the predominant bacterial species. Genomic analysis showed that bacteria isolated from secondary infection had high homology with the corresponding predominant intestinal opportunistic pathogens. In addition, animal model experiments validated the hypothesis that the administration of antibiotics caused the enrichment of CRKP and VRE among the intestinal microbiota, increasing the likelihood of permeation of other tissues and potentially causing subsequent systemic infection in pseudo germ-free mice.

**Conclusion:**

Our study indicated that the pathogens causing secondary infection in septic patients might originate from the intestinal colonization of pathogens following broad-spectrum antibiotic treatment.

**Supplementary Information:**

The online version contains supplementary material available at 10.1186/s13054-022-03943-z.

## Take home message

1. The gut microbiota of septic patients tends to be dominated by *Klebsiella* or *Enterococcus* after broad-spectrum antibiotic treatment.
2. Intestinal *Klebsiella* and *Enterococcus* might translocate from the gut to the bloodstream or pulmonary system, leading to systemic infection and increasing the risk of mortality in septic patients

## Introduction

Sepsis, a clinical syndrome occurring in patients following infection or injury [1], is one of the leading causes of mortality in the intensive care unit (ICU) [2]. Previous reports indicated that sepsis-associated mortality is frequently attributed to subsequent secondary nosocomial infection and multiple organ dysfunction syndrome (MODS). Among the complicated factors related to the pathogenesis, gut microbiota dysbiosis is widely believed to play a crucial role in secondary nosocomial infection development [3]. A large number of alterations due to the onset of sepsis alters gut integrity and increases intestinal permeability, which has been speculated to lead to bacterial translocation through the mesenteric lymph nodes or portal venous blood and induce associated inflammatory response syndrome (SIRS) and MODS [3, 4].

A balanced microbiota plays an important role in the digestion of food, protection against epithelial cell injury, development of the immune system, and resistance to colonization by pathogens [5]. The usage of broad-spectrum antibiotics and dysbiosis of the gut microbiota in critically ill patients may hinder host innate immune defenses against infection, leading to susceptibility to intestinal colonization of vancomycin-resistant *Enterococcus faecium* (VRE) and the development of invasive bloodstream infection [6]. Gut microbiota dysbiosis is characterized as disorder in the composition of the intestinal microbiota [4]. Compared to that of healthy controls, the gut microbiota of septic patients is characterized by less diversity, lower abundances of key commensal genera and sometimes the overgrowth of a single species, such as *Clostridium difficile*, *Salmonella spp*., *Staphylococcus* spp., and *Enterococcus *spp.[7]. Modulation of the intestinal microbiota is beneficial in decreasing the risk of sepsis-related mortality [3]. A previous study revealed that certain commensal gut microbes increased the level of serum immunoglobulin A antibodies, which protect the intestines from polymicrobial sepsis [8]. The normal intestinal microbiota promotes postnatal granulocytosis and IL-17-dependent host resistance to sepsis in neonatal mice [9].

In this prospective observational study, we detected the phylogenetic composition of the gut microbiota at multiple time points in stool samples from septic and non-septic ICU patients using 16S rRNA gene amplicon sequencing, aiming to characterize the transformation of the gut microbiota in septic patients and potential influencing factors. Then, we performed a genomic analysis to compare intestinal and systemic pathogenic bacteria and investigate their homology. Moreover, we established pseudo germ-free mice and challenged them with exogenous *Klebsiella pneumoniae* to verify that secondary infectious bacteria might originate from the predominant opportunistic intestinal pathogens.

## Materials and methods

### Patient enrollment

From March 2020 to September 2020, every consecutive patient admitted to the emergency ICU of Zhongshan Hospital, Fudan University, Shanghai, China, was prospectively enrolled. The diagnosis of sepsis was based on The Third International Consensus Definitions for Sepsis and Septic Shock (Sepsis-3), and suspected infection was defined as a Sequential Organ Failure Assessment (SOFA) score ≥ 2 [10]. Thirty-four patients with sepsis and thirty-three non-septic patients were enrolled in this study. Non-septic patients in critical condition who were admitted to the ICU were recruited as ICU controls; the diagnoses of the ICU control patients included acute heart failure (*n* = 12), asthma (*n* = 5), pneumothorax (*n* = 6) and hypertensive emergencies (*n* = 10). Ten healthy, nonsmoking human subjects who had not taken antibiotics during the previous year served as healthy controls.

**Fig. 1:**
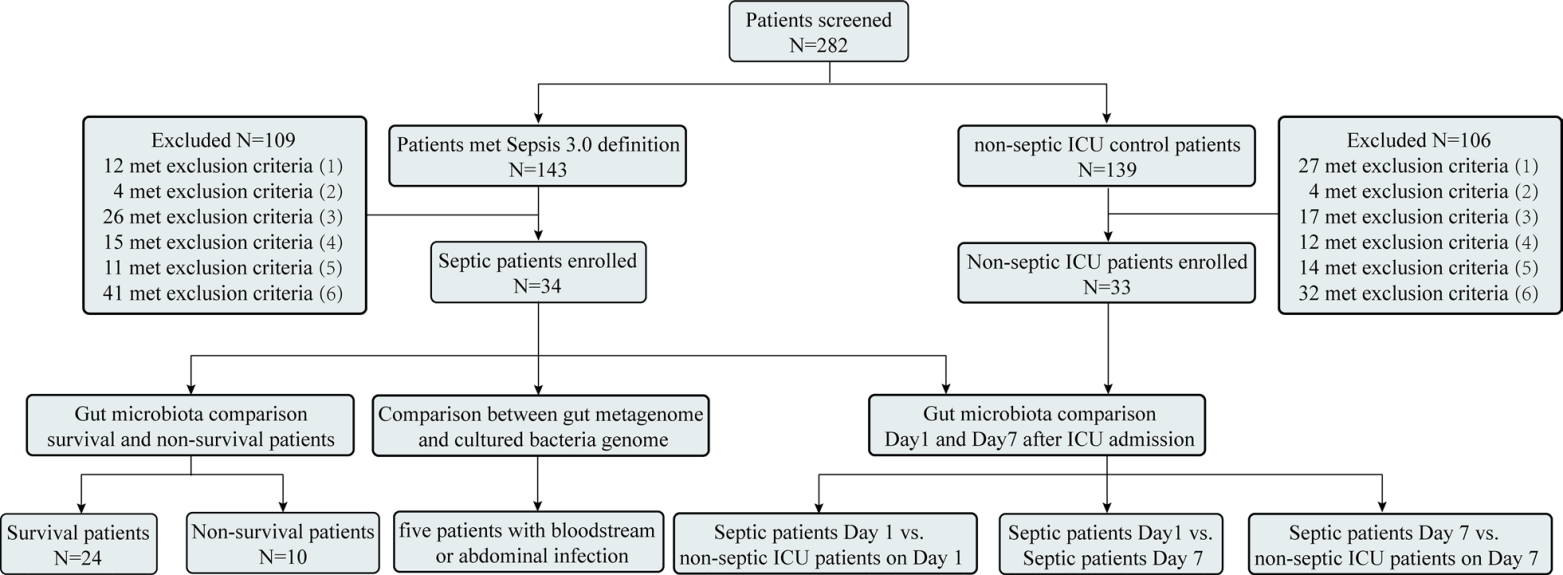
Flow chart of patients enrolled

The exclusion criteria were as follows: (1) patients diagnosed with intestinal dysfunction (Crohn's disease, ulcerative colitis, irritable bowel syndrome, etc.); (2) age < 18 years or pregnancy; (3) long-term immunosuppression or organ transplantation; (4) transfer from another ICU; (5) length of ICU stay < 7 days; and (6) tumor or antibiotic treatment within 180 days. A flowchart illustrating the recruitment process is shown in Fig. 1.

### Secondary infection

Secondary infection was diagnosed according to the Centers for Disease Control and Prevention’s National Healthcare Safety Network (CDC/NHSN) Surveillance Definition of Health Care-Associated Infection and Criteria for Specific Types of Infections in the Acute Care Setting [11]. Only new-onset nosocomial infection identified at more than 48 h after ICU admission was classified as secondary infection. The time of secondary infection was the day when positive cultures were collected or when signs/symptoms developed if no positive cultures were obtained. Three experienced doctors were responsible for the diagnosis of secondary infection.

### Data collection at inclusion

The following demographic, clinical and biochemical data were collected on Day 1 and Day 7 after ICU admission: age, sex, Acute Physiology and Chronic Health Evaluation II (APACHE II) and SOFA scores, mechanical ventilation, vasoactive agent use, characteristics of infection if relevant (site, method of detection, and identified organisms), antibiotic treatment, etc. The SOFA and APACHE II scores were calculated based on daily clinical indicators using previously described methods [12, 13].

### Sample collection

Fresh stool samples were collected from patients and healthy volunteers and stored at −80 ℃. Fresh mouse stool pellets were obtained before the mice were euthanized, and blood was collected from the orbital vein of the mice in sterile anticoagulant tubes. The inferior lobes of the right lung were excised, placed in tubes containing 1 ml of sterile PBS and homogenized mechanically for the colony forming units (CFU) assay. The superior lobes of the right lung were collected for immunofluorescence staining, and the rest of the lung tissue was collected for 16S rRNA gene amplicon sequencing. The contents of the distal 3 cm of the colon were removed by manual extrusion, and the tissues were placed in 4% paraformaldehyde for immunohistochemical analysis.

### Bacterial culture of blood, peritoneal fluid and bronchoalveolar lavage fluid samples from septic patients

Samples were selected with inoculating loops and spread on LB agar plates. After incubation at 37 ℃ for 16 h, a single colony was placed in LB medium and incubated in a shaker at 37 ℃ and 220 rpm. The bacteria were mixed in a 1:1 (v: v) ratio with a sterile 50% glycerin solution and preserved at −80 ℃.

### DNA extraction, PCR amplification and 16S rRNA gene amplicon sequencing

Genomic DNA was extracted from stool, lung tissue, and blood samples using a QIAamp DNA Stool Mini Kit (Qiagen, Hilden, Germany) according to the manufacturer’s instructions. The integrity and size of genomic DNA were assessed using 1.5% agarose gel electrophoresis. Primers with barcodes designed to target the V3-V4 hypervariable region were used to generate 16S rDNA gene amplicons. All amplicons were purified with a QIAquick PCR Purification Kit (Qiagen, Hilden, Germany), quantified on a Qubit instrument (Life Technologies, New York, US), and then pooled at equal concentrations. Paired-end sequencing (2 × 300 bp) was performed on pooled amplicons using the Illumina MiSeq platform.

### Genome sequencing and assembly

Genomic DNA was extracted from cultured *Klebsiella pneumoniae* and *Enterococcus faecium* strains using the QIAamp DNA Mini Kit (Qiagen, Hilden, Germany). A 300-bp paired-end library was constructed for purified bacterial DNA samples and stool DNA samples using the standard Illumina paired-end protocol. Cluster generation was performed in C-bot, and 2X 150-bp paired-end sequencing was performed using Illumina X10 instrument (Illumina, San Diego, CA, USA). Bacterial genome assembly was performed using the program Velvet 1.2.10 [14]. The metagenomic DNA of stool samples was assembled using SPAdes-3.12.0 [15]. The clean reads of metagenomic sequences were mapped to the reference genome using Bowtie2 (-very-fast-local) [16]. The mapping results were processed with SAM tools. Raw SNPs were called using GATK HaplotypeCaller.

### Mouse models and husbandry conditions

Six-week-old female C57BL/6J mice were purchased from Shanghai Jie Si Jie Laboratory Animal Co. Ltd. The mice were housed in a humidified room with a 12:12 h light–dark cycle and were provided with irradiated food and sterile water at the Department of Laboratory Animal Science, Zhongshan Hospital, Fudan University, China. For experiments involving antibiotic treatments, broad-spectrum antibiotics (ampicillin 1 g/L, Macklin; neomycin sulfate 1 g/L, Macklin; metronidazole 1 g/L, Macklin; and vancomycin 0.5 g/L, CSNpharm) were administered in the drinking water for two weeks [17]. All experiments involving live animals were conducted in compliance with the ‘Guide for the Care and Use of Laboratory Animals’ and were approved by the Institutional Review Board of Zhongshan Hospital, Fudan University, China (No: 201804001Z).

#### *Klebsiella* infection

Mice were infected with *Klebsiella pneumoniae* by gavage with 5 × 10^6^ CFUs of *Klebsiella* in 200 μL of Luria–Bertani medium. *Klebsiella* colonies were identified based on their appearance and confirmed by plating on a KPC culture plate (CHROMagar, France). After infection, animals were euthanized at different time points: 24 h, 48 h, and 72 h. Lung tissue, colon tissue, feces, and blood were collected from mice.

#### Lung and blood CFU assay

Aliquots of lung homogenates and blood in tenfold dilutions were plated onto KPC media and incubated aerobically at 37 °C. Colonies were counted after 16 h of growth.

#### Intestinal barrier integrity

The integrity of the intestinal barrier was assessed using the FITC-dextran assay, as described previously [18]. Mice were treated with 200 μl of FITC-dextran at 600 mg/kg, p.o. Four hours later, blood was collected by eyeball extirpation. FITC-dextran was diluted in saline to construct a standard curve, all samples were analyzed at an excitation wavelength of 480 nm, and measurements were recorded at an emission wavelength of 520 nm.

#### Bioinformatics and statistical analyses

Categorical variables are presented as N and %, and Fisher’s exact test or the *χ*^2^ test was used to compare data. Continuous variables are presented as means ± standard error of the means (SEMs) or medians (interquartile ranges [IQRs]) and were compared using Student’s *t* test or the Mann–Whitney *U* test, as appropriate. A paired *t* test was used to compare the parameters of patients with sepsis on Day 1 and Day 7. Data were analyzed using SPSS version 22.0 (IBM), and statistical charts were generated using Prism 7.0 software (GraphPad). For all statistical analyses, *P* < 0.05 was considered significant.

The paired-end 16S rDNA sequences were assembled using Mothur (version 1.41.1) [19]. DNA sequences were filtered using the following criteria: ambiguous bases, chimeric or contaminant sequences, and lengths shorter than 350 bp. Sequence alignment was performed using the SILVA reference database (V132) [20]. DNA sequences were clustered into operational taxonomic units (OTUs) at 97% similarity. After data normalization, assessments of community richness, evenness, and diversity (Shannon, Simpson, Shannoneven, Simpsoneven, ACE, and Chao index and Good’s coverage) were also performed using Mothur. The online software RDP classifier (80% threshold) [21] assigned the sequences to each OTU based on the Ribosomal Database Project [22]. A representative sequence of each OTU was used as a query sequence to define species through a BLASTN search against the NCBI database with more than 99% identity and the highest total score. Differences in bacterial diversity were assessed using analysis of molecular variance (AMOVA). The differences in features (taxonomy and OTUs) were determined using STAMP (*P* < 0.05, difference between proportions > 0.5%) [23]. The correlation coefficients between bacterial genera and factors were calculated with R using the nonparametric Spearman rank correlation algorithm; a coefficient of > 0.68 or < −0.68 was considered to represent a strong correlation [24]. Multivariate linear regression models were used to control the potential confounding factors such as disease severity that might affect the gut microbiota difference between septic patients and non-septic patients. Alpha diversity indexes including ACE, Chao, Simpson and Shannon were log transformed as dependent variable and SOFA and APACHE II scores were included in the regressions.

## Results

### Characteristics of the enrolled patients

Three hundred and twelve fecal samples were collected from 34 patients with sepsis (age 58.50 years, IQR 58.00–67.25 years, 64.70% male) and 33 non-septic patients (ICU controls) (age 69.00 years, IQR 50.00–76.00 years, 54.54% male) between 1 and 47 days after ICU admission. We also collected fecal samples from 10 healthy individuals as additional controls. The characteristics of all the enrolled patients are listed in Additional file 1: Table S1. The 60-day mortality of septic patients was 29.41% (10 in 34), while that in the non-septic ICU patients was 2.94% (1 in 33) (*P* < 0.01). In total, 23 (67.64%) septic patients had secondary infections in various organs, including the lungs (*n* = 16), urinary system (*n* = 11), bloodstream (*n* = 9) and abdomen (*n* = 1), while only two non-septic ICU controls acquired secondary infections (*P* < 0.01). Fourteen septic patients (14/23, 60.87%) had secondary infections caused by *Klebsiella pneumoniae,* and six of them died. Among the nine septic patients with bloodstream infections, seven were infected with CRKP (Additional file 2). All these results suggested that *Klebsiella pneumoniae* was the most common and lethal pathogen causing nosocomial infection.

### *Carbapenem-resistant Klebsiella pneumoniae* predominated the intestinal microbiota in septic patients

#### Genomic analysis of the gut microbiota of septic patients on the day of ICU admission

**Fig. 2:**
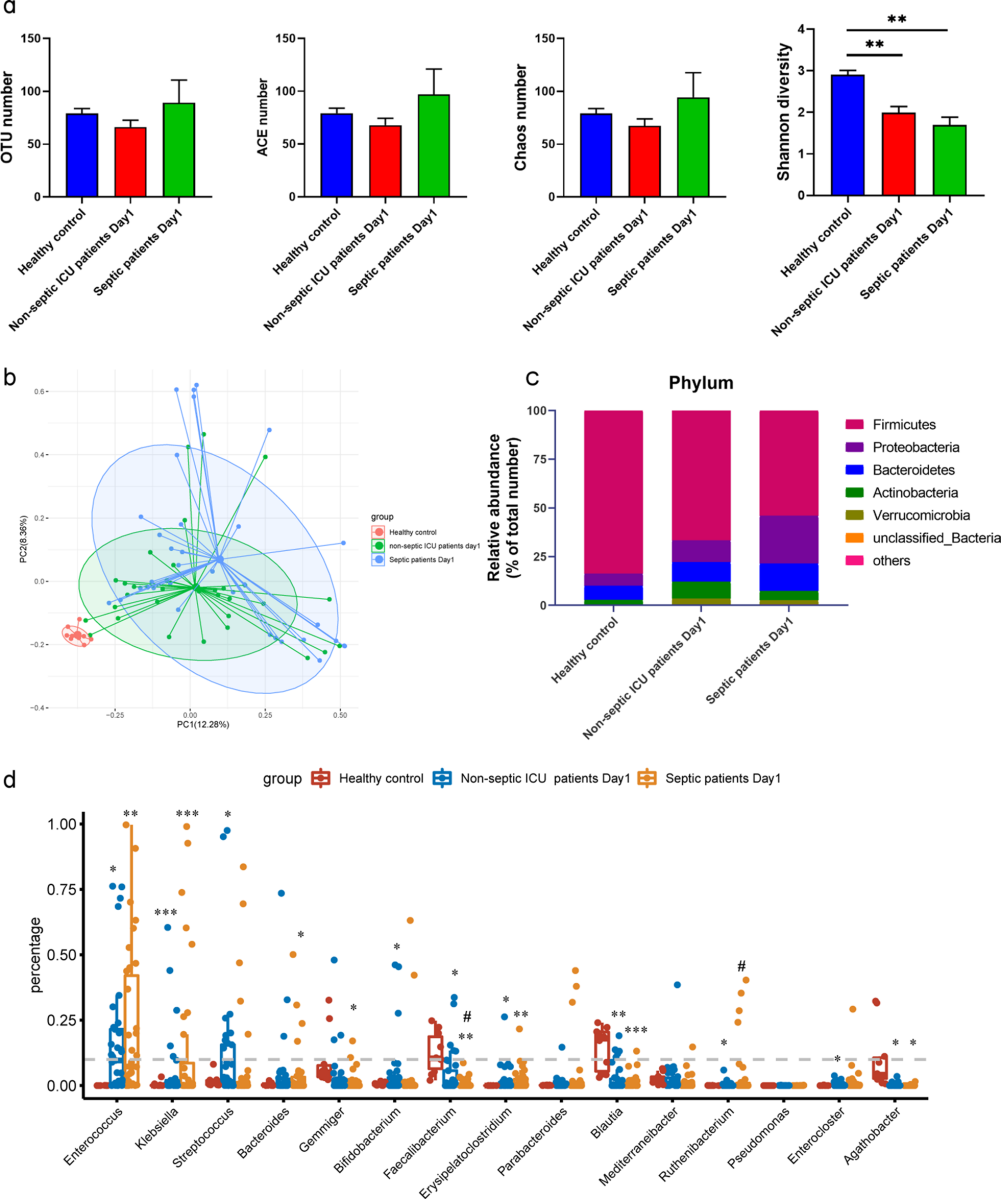
Alterations in the gut microbiota between septic and non-septic ICU patients on day 1 after ICU admission. **a.** Alpha diversity analysis based on number of OTUs and ACE and Chao indices and beta diversity analysis based on the Shannon diversity index among the three groups. **b.** Principal coordinates analysis (PCoA) using unweighted UniFrac distances showed significant differences in the microbiota composition of the three groups. **c**. Mean proportions of phylum compositions in the three groups. **d.** Proportions of genera of the three groups. *indicates comparisons with healthy controls, # indicates comparisons with septic patients on Day 1. *P < 0.05, **P < 0.01, ***P < 0.001, and ^#^*P* < 0.05

On the first day of ICU admission, septic patients had a greater severity of illness and stronger inflammatory responses than ICU control patients. As illustrated in Table 1 (Septic patients, Day 1 *vs.* non-septic ICU patients, Day 1), septic patients had higher SOFA and APACHE II scores (*P* < 0.001), along with higher levels of C-reaxctive protein (CRP), procalcitonin (PCT), tumor necrosis factor (TNF)-α, interleukin (IL)-2R, IL-6, IL-8 and IL-10 than non-septic ICU controls. In addition, the Shannon diversity indices for both septic and non-septic patients were significantly lower (*P* < 0.01) than that for healthy controls on the ICU admission day (Fig. 2a). Principal coordinates analysis (PCoA) of all samples indicated a clear partitioning between healthy controls and septic/non-septic patients, with a value of 20.64% (*P*_AMOVA_ < 0.001), but no difference was observed between septic and non-septic patients (*P*_AMOVA_ = 0.096) (Fig. 2b).

**Table 1:** Clinical characteristic of septic and non-septic ICU patients on admission and 7th day in ICU

	Day1			Day7			Septic patients Day1 vs septic patients Day7 *P* value
	Septic patients (n = 34)	non-septic ICU patients (n = 33)	*P* value	Septic patients (n = 34)	non-septic ICU patients (n = 33)	*P* value	
SOFA score	4.5 (4.0, 8.0)	2.0 (0.0, 2.0)	< 0.001	5.0 (3.0, 7.3)	1.0 (0.0, 2.0)	< 0.001	0.848
APACHE II score	13.5 (8.8, 19.5)	7.0 (3.0, 11.0)	< 0.001	11.5 (7.0, 19.3)	6.0 (3.0, 8.0)	< 0.001	0.253
WBC (10^9^/L)	10.9 (6.6, 15.3)	8.0 (5.5, 11.25)	0.077	10.6 (5.5, 16.38)	7.0 (4.9, 8.9)	0.013	0.941
CRP (mg/L)	104.0 (54.8, 204.7)	51.3 (5.0, 11.3)	0.011	32.6 (12.4, 72.0)	9.5 (5.0, 32.8)	0.003	< 0.001
PCT (ng/ml)	1.8 (0.5, 16.0)	0.2 (0.11, 0.71)	< 0.001	0.6 (0.2, 2.2)	0.2 (0.1, 0.4)	0.001	0.015
TNF-α (pg/L)	15.1 (9.5, 23.8)	11.8 (7.0, 18.8)	0.044	13.6 (9.3, 22.6)	7.5 (5.3, 14.9)	0.024	0.522
IL-1β (pg/L)	5.0 (5.0, 5.0)	5.0 (5.0, 5.0)	0.801	5.0 (5.0, 5.0)	5.0 (5.0, 5.2)	0.552	0.94
IL-2R (pg/L)	1519.0 (940.0, 1931.0)	567.0 (416.0, 812.0)	< 0.001	1070.0 (694.5, 1310.0)	528.5 (413.8, 651.5)	0.003	0.011
IL-6 (pg/L)	36.1 (14.4, 89.6)	15.9 (7.5, 36.1)	0.016	18.75 (6.7, 47.0)	8.1 (4.8, 15.8)	0.082	0.027
IL-8 (pg/L)	46.0 (22.0, 90.0)	21.0 (9.0, 44.0)	0.007	34.5 (16.3, 61.5)	15.0 (11.3, 24.0)	0.009	0.228
IL-10 (pg/L)	13.1 (6.7, 31.9)	5.0 (5.0, 6.8)	< 0.001	6.8 (5.0, 15.8)	5.0 (5.0, 6.4)	0.004	0.032

SOFA, Sequential Organ Failure Assessment; APACHE II, Acute Physiology and Chronic Health Evaluation II; WBC, white blood cell; CRP, C-reactive protein; PCT, procalcitonin; TNF-α, tumor necrosis factor-α; IL-1β, interleukin-1β; IL-2R, interleukin-2 receptor; IL-6, interleukin-6; IL-8, interleukin-8; IL-10, interleukin-10.

A larger number of antibiotics and broad-spectrum antibiotics were administered to patients with sepsis after admission than to ICU control patients (Additional file 1: Figure S1c). The gut microbiota was altered by antibiotic treatment, and taxonomic analysis revealed that the abundance of potential pathogens was indeed increased and the abundance of some probiotics was decreased in septic patients. The gut microbiota of healthy controls was predominantly populated by *Firmicutes* (83.73%), *Bacteroidetes* (7.20%), and *Proteobacteria* (6.32%) at the phylum level (Fig. 2c). The proportion of *Firmicutes* was significantly lower in non-septic ICU patients (66.71%, *P* = 0.02) and septic patients (54.09%, *P* < 0.001) than in healthy controls (Fig. 2c). However, the proportions of *Bacteroidetes* and *Proteobacteria* were significantly higher in septic and non-septic patients than in healthy controls, including pathogenic and lipopolysaccharide-containing bacteria, such as *Escherichia* and *Klebsiella*. In addition, the proportion of *Proteobacteria* in septic patients (24.48%) was significantly higher than that in non-septic patients (11.28%). Twenty-seven genera in the microbiome showed significant differences among the three groups, of which the top 15 genera are illustrated in Fig. 2d. *Enterococcus* and *Klebsiella*, the top two genera, had significantly higher percentages in both septic and non-septic ICU patients than in healthy controls. The average proportion of *Klebsiella* in septic patients was higher than that in ICU controls. Moreover, the proportions of *Faecalibacterium* and *Blautia* in septic and non-septic ICU patients were significantly lower than those in healthy controls.

#### *Klebsiella pneumoniae* became the dominant species in the gut microbiota of septic patients one week after ICU admission

**Fig. 3:**
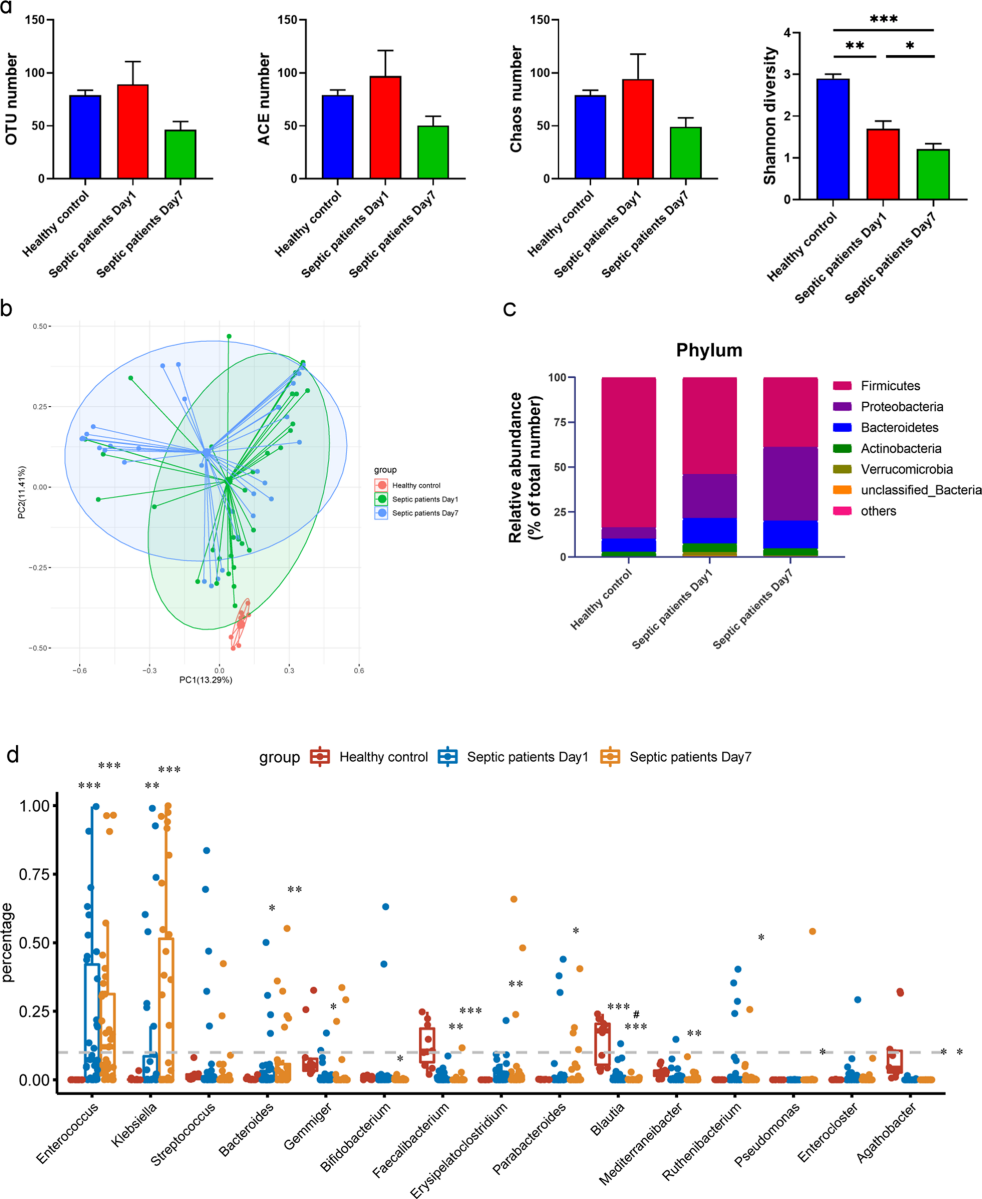
Alterations in the gut microbiota of septic patients on Days 1 and 7 after ICU admission. **a**. Alpha diversity analysis based on the number of OTUs and ACE and Chao indices and beta diversity analysis based on the Shannon diversity index among the three groups. **b.** Principal coordinate analysis (PCoA) using unweighted UniFrac distances showed significant differences in the microbiota compositions of the three groups. **c**. Mean proportions of phylum compositions in the three groups. **d**. Proportions of genera in the three groups. *indicates comparisons with healthy controls, ^#^ indicates comparisons with septic patients on Day 1. *P < 0.05, **P < 0.01, ***P < 0.001, and ^#^*P* < 0.05

After one week of hospitalization, septic patients showed a clear decrease in serum levels of CRP, PCT, IL-2R, IL-6 and IL-10 (Table 1, Septic patients Day1 *vs.* Septic patients Day 7). Following treatment with broad-spectrum antibiotics (Additional file 3) and other interventional therapies, the Shannon diversity index of the gut microbiota of septic patients was remarkably decreased on the 7th day compared to that on the day of ICU admission (Fig. 3a). At the phylum level, the mean proportion of *Firmicutes* showed a continuous decrease in patients with sepsis from 54.09% on Day 1 to 38.92% on Day 7 (Fig. 3c, *P* < 0.001). Further analysis indicated that with the progression of sepsis, the proportions of opportunistic genera, including *Klebsiella* (*P* = 0.007), *Bacteroides* (*P* = 0.03) and *Erysipelatoclostridium* (*P* = 0.007), increased significantly in septic patients (Fig. 3d). Notably, the proportion of *Klebsiella* in the gut microbiota of septic patients on Day 7 was significantly higher than that in healthy controls and septic patients on Day 1 (Fig. 3d, *P* < 0.001). Based on the aforementioned factors, the relative abundance of *Klebsiella* continued to increase and became the dominant genus in septic patients after one week in the hospital (Fig. 3d). According to these results, the composition of the intestinal microbiota changed drastically during the progression of sepsis and broad-spectrum antibiotic use, and such changes were mainly characterized by the decreased abundance of probiotic species and the increased abundance of opportunistic pathogens, such as *Klebsiella*.

#### The gut microbiota alterations between septic and non-septic ICU patients after one-week hospitalization

We further analyzed clinical indicators in all patients on day 7 of ICU admission and found that the SOFA and APACHE II scores and inflammatory indicators in septic patients were significantly higher than those in ICU controls (Table 1, Septic patients Day 7 vs. non-septic ICU patients Day 7). Then, we analyzed the gut microbiota composition and found that the Shannon diversity index was significantly lower in septic patients than in ICU and healthy controls (Additional file 1: Figure S2a). PCoA indicated clear partitioning among healthy controls, ICU controls and septic patients, with a value of 25.03% (Additional file 1: Figure S2b, *P*_ANOSIM_ < 0.001).

On day 7 of ICU admission, the proportion of *Firmicutes* declined to 38.92%, while that of *Proteobacteria* increased to 40.96% in septic patients (Additional file 1: Figure S2c). At the genus level, the intestinal microbiota of most septic patients was dominated by *Klebsiella*, and the proportion was significantly higher than that in non-septic ICU patients (Additional file 1: Figure S2d, *P* < 0.01). Consistent with the results on day 1, the proportions of *Klebsiella* in septic and ICU control patients were significantly higher than that in healthy controls (Additional file 1: Figure S2d, *P* < 0.001). Similarly, the proportion of *Enterococcus* was increased in septic patients, and the proportion was higher than that in ICU control patients.

Though septic patients showed more severe illness than non-septic ICU patients at Day 1, the diversity of gut microbiota had no significant difference between the two groups (Fig. 2a). But after one-week broad-spectrum antibiotics treatment for septic patients, the diversity of gut microbiota in septic patients was significantly reduced compared with non-septic patients at Day 7 (controlled for SOFA and APACHE II scores using multivariate linear regression models) (Additional file 1: Table S2).

#### Differences in the gut microbiota between survival and non-survival septic patients

Ten septic patients died within 60 days after admission (detailed clinical information is listed in Additional file 1: Table S3 and Additional file 2). A higher percentage of non-survival patients had bloodstream infections than survival patients. No significant differences in clinical indicators were observed between days 1 and 7 among deceased patients with sepsis (Additional file 1: Table S4), while among survival septic patients, indicators such as the APACHE II score and the levels of CRP, PCT, IL-2R and IL-10 were all decreased after treatment (Additional file 1: Table S5). Moreover, the number of OTUs and the ACE, Chao, Shannon diversity indices of the gut microbiota on Day 7 in non-survival patients were all significantly decreased compared with those on Day 1 (Additional file 1: Figure S3a). However, the gut microbiota was not significantly altered at the phylum or genus level during the treatment period in deceased septic patients (Additional file 1: Figure S3b-d). The number of OTUs and the ACE, Chao, Shannon diversity indices remained relatively stable during the one-week hospitalization period in survival patients (Additional file 1: Figure S4a). In addition, the relative abundance of the phylum *Proteobacteria* was increased, while the abundances of eight anomalous genera were reduced to a relatively normal level (Additional file 1: Figure S4b–d).

### Correlation between the microbiota composition and clinical indicators

**Fig. 4:**
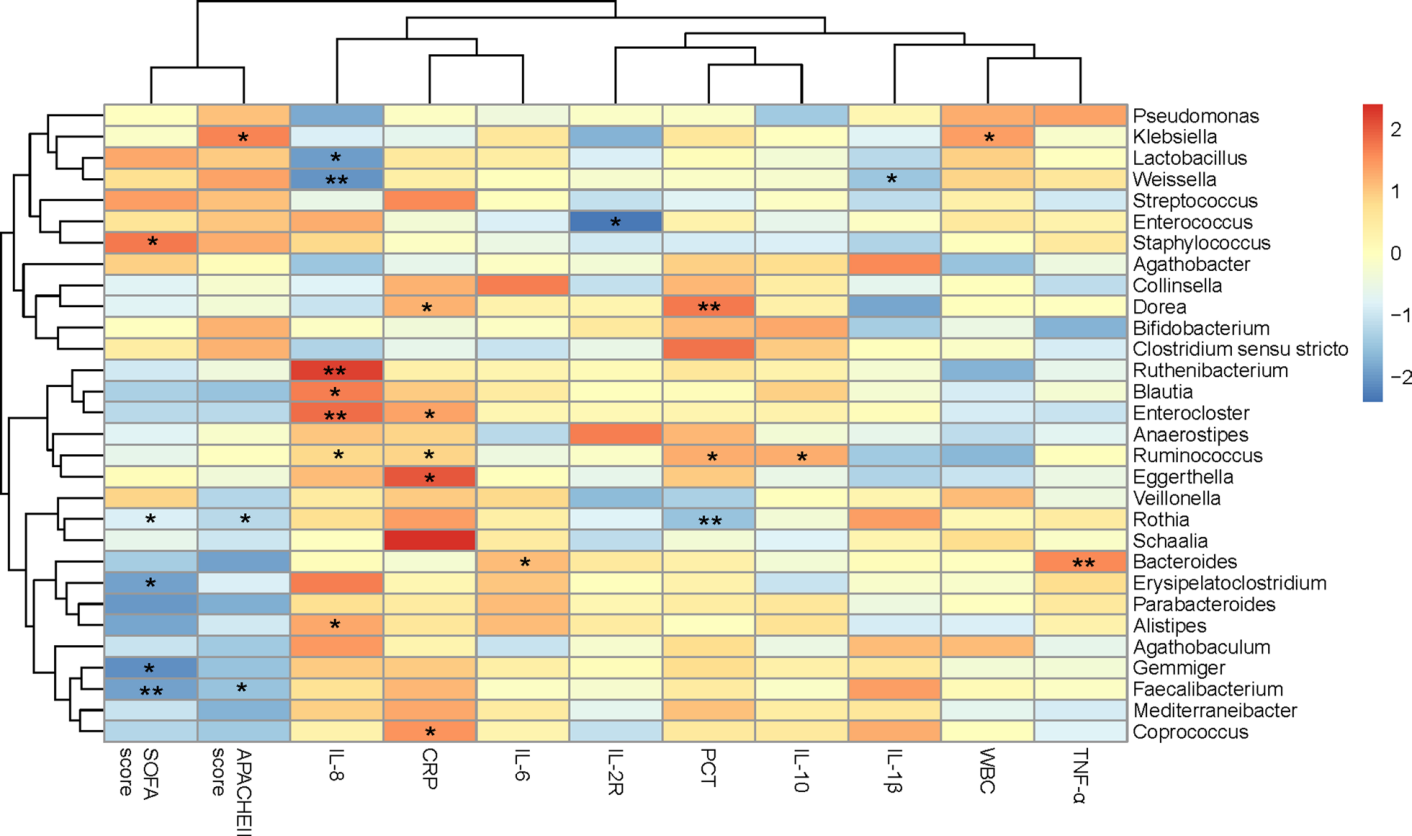
Correlation between clinical indicators and the gut microbiota composition at the genus level in septic patients. The red color represents a positive correlation, and the blue color represents a negative correlation. The deeper the color is, the higher the R value. *P < 0.05 and **P < 0.01

We performed Spearman’s correlation analysis to analyze associations of patient clinical indicators and the top 30 microbial genera at specific corresponding time points to determine whether the relative abundances of members of the gut microbiota were associated with any clinical parameter (Fig. 4). The changes in 19 genera were significantly correlated with the indicators over time. As illustrated in Fig. 4, the SOFA score was positively correlated with the relative abundance of *Streptococcus* (*P* < 0.05, rho = 0.189) and negatively correlated with the abundance of *Faecalibacterium* over time (*P* < 0.01, rho = -0.237). The APACHE II score showed a positive correlation with *Klebsiella* (*P* < 0.05, rho = 0.191) and negative correlations with *Rothia* (*P* < 0.05, rho = -0.214) and *Faecalibacterium* (*P* < 0.05, rho = -0.190). Moreover, *Lactobacillus* abundance was negatively correlated with serum IL-8 levels (*P* < 0.05, rho = − 0.237). Detailed information is listed in Additional file 1: Table S6.

### Temporal changes in the related bacteria involved in intestinal-dominant and secondary systemic infections

**Fig. 5:**
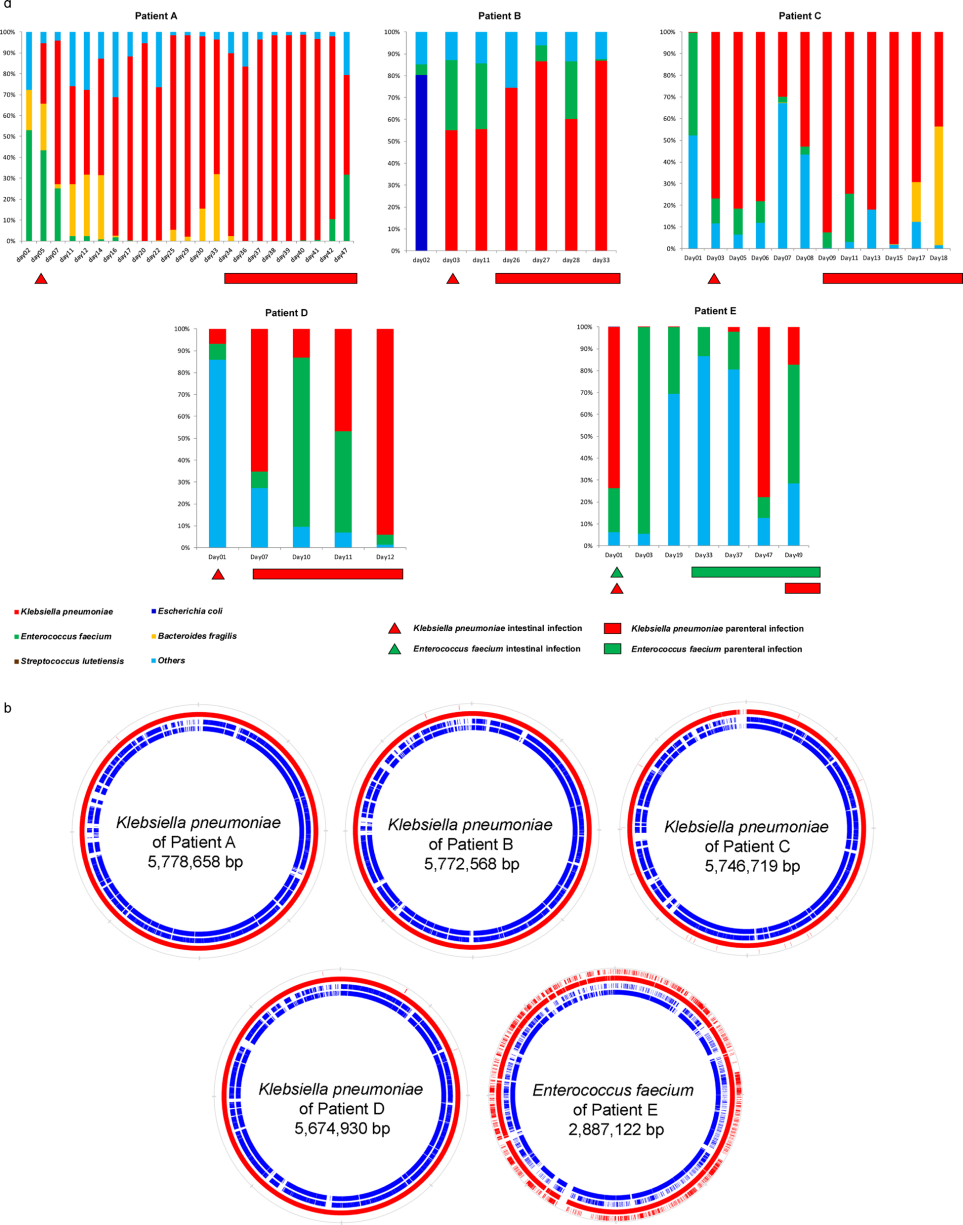
**a**. Time points of intestinal and secondary infections. The bar chart represents the microbiome composition at the genus level at different time points for individual patients. The triangle indicates the time of *Klebsiella* or *Enterococcus* intestinal predominance, and the bar indicates the secondary infection time. In Patient A, the intestinal *Klebsiella* predominance time was Day 5, and the secondary bloodstream infection time was Day 32. In Patient B, the intestinal *Klebsiella* predominance time was Day 3, and the systemic bloodstream infection time was Day 26. In Patient C, the intestinal *Klebsiella* predominance time was Day 3, and the secondary bloodstream infection time was Day 6. In Patient D, the intestinal *Klebsiella* predominance time was Day 1, and the secondary bloodstream infection time was Day 2. In Patient E, the intestinal *Klebsiella* predominance time was Day 1, and the secondary bloodstream infection time was Day 47. In Patient E, the intestinal *Enterococcus* predominance time was Day 5, and the secondary abdominal infection time was Day 24. **b**. Comparison of the intestinal metagenomic sequences and cultured bacteria sequences from patients. Using the concatenated draft genome of cultured bacteria from each patient as a reference, the intestinal metagenomic sequences were mapped to the reference, and SNPs are illustrated in the first (outer) circle. The second circle represents the region of the reference genome covered by the metagenome sequences (red). The third and fourth circles (innermost) show the genome comparison result for one randomly downloaded complete genome from NCBI with the reference draft genome (blue), with the third circle representing SNPs and the fourth circle representing coverage. The gaps in the circles indicate that this region was not covered by a metagenomic sequence (the second circle) or other known genomes (the fourth circle)

*Klebsiella pneumoniae* and *Enterococcus faecium* are the most common pathogens causing secondary or nosocomial infection in septic patients with prolonged ICU stays. Multiple fecal samples were periodically collected from five septic patients who developed nosocomial bloodstream or abdominal infection during hospitalization (Fig. 5a). Patients A–D developed *Klebsiella* bloodstream infection, while Patient E developed *Klebsiella* and *Enterococcus* abdominal coinfection. The intestinal microbiota of Patient A was altered substantially during hospitalization, and *Klebsiella pneumoniae* eventually became the dominant organism on Day 5. This shift preceded the detection of the *Klebsiella* bloodstream infection by 29 days. Patients B and C exhibited similar alterations in the fecal microbiota: *Klebsiella pneumoniae* was first detected in the gut on Day 3 and then in the bloodstream on Day 13 in Patient B and on Day 9 in Patient C. The secondary infection site was the bloodstream, and a six-day interval between the detection of *Klebsiella pneumoniae* predominance in the gut and bloodstream infection was observed in Patient D. On the day of admission, *Klebsiella pneumoniae* was the dominant organism in fecal samples from Patient E; however, no corresponding systemic infection was observed on several subsequent days of hospitalization. *Enterococcus faecium* became the dominant organism on the 3rd day and an *Enterococcus* abdominal infection developed on the 24th day after admission. *Klebsiella pneumoniae* became dominant again on the 47th day after admission, and two days later, an abdominal *Klebsiella pneumoniae* infection was detected. These findings revealed that secondary infection caused by highly antibiotic-resistant bacteria occurred after intestinal domination.

### Homology analysis of intestinal and secondary infectious organisms

We performed a homology analysis to compare secondary infection pathogens and dominant intestinal bacteria to determine whether *Klebsiella pneumoniae* and *Enterococcus faecium* in fecal samples were the sources of the pathogens identified in blood and ascites. Four fecal samples from *Klebsiella pneumoniae*-infected patients (Patients A–D) and one *Enterococcus faecium*-infected patient (Patient E) were sequenced, and the reads were mapped to the genome sequences of cultured *Klebsiella pneumoniae* and *Enterococcus faecium* originating from these patients, respectively (Fig. 5b). Only one, five, 15 and 21 single nucleotide polymorphisms (SNPs) were identified in the comparison between the intestinal metagenome and cultured *Klebsiella pneumonia* genome from bloodstream (Fig. 5b, Patients A–D), and 3,692 SNPs were identified in the comparison of the intestinal metagenome and cultured *Enterococcus faecium* genome from bloodstream samples of Patient E (Fig. 5b, Patient E), indicating more than 99.87% homology. In addition, all contigs belonging to plasmids of *Klebsiella pneumoniae* and *Enterococcus faecium* were covered by metagenomic sequences, indicating that *Klebsiella pneumoniae* and *Enterococcus faecium* in the gut might be sources of blood and abdominal infections. Moreover, two *K. pneumoniae* strains were separately isolated from the lung of Patients B and D, and their genomic sequences also showed high homology with metagenomic sequences (Additional file 1: Fig. 5). These findings indicated high homology between intestinal and secondary *Klebsiella pneumoniae* and *Enterococcus faecium*.

### Protective effect of the gut microbiota on intestinal *Klebsiella* infection

**Fig. 6:**
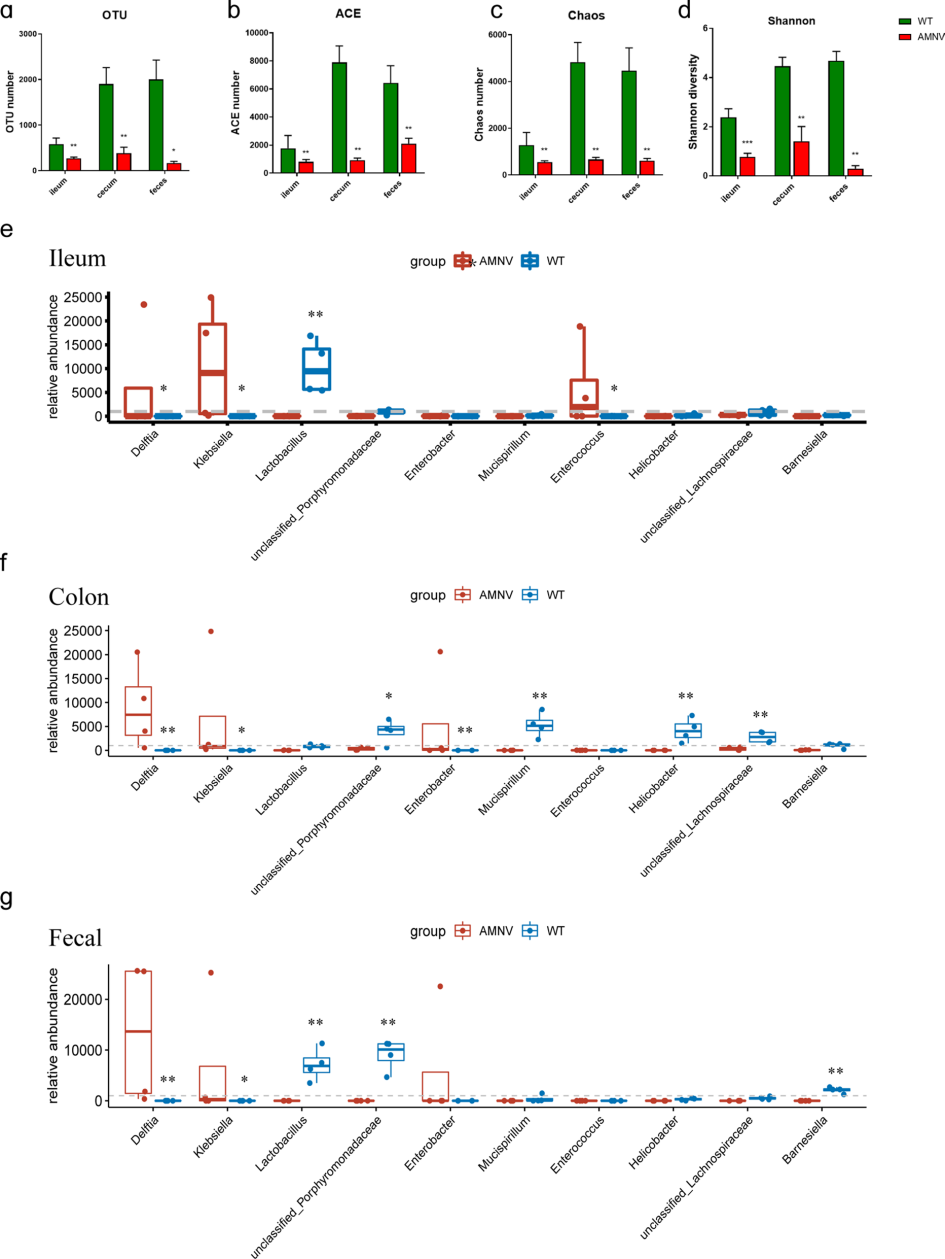
General alterations in the gut microbiota in different intestinal segments after antibiotic administration. **a–d**. Alpha diversity analysis based on the number of OTUs and ACE and Chao indices and beta diversity analysis based on the Shannon diversity index in different intestinal segments between AMNV-treated and WT mice. **e–g**. Proportions of genera in different intestinal segments of AMNV-treated and WT mice. *indicates comparisons with AMNV-treated mice. *P < 0.05 and **P < 0.01.

The aforementioned results indicated that *Klebsiella pneumoniae* infection was one of the main factors contributing to mortality among septic patients. We administered broad-spectrum antibiotics (ampicillin, metronidazole, neomycin sulfate, and vancomycin [AMNV]) to wild-type (WT) mice in drinking water for two weeks to deplete the gut microbiota and then challenged them with *Klebsiella pneumoniae* to explore the relationship between opportunistic pathogen colonization in the intestine and systemic infection. As indicated in Fig. 6a-d, the relative abundance of bacteria in the gut microbiota, detected in the feces, as well as colonization of the ileum and cecum, were all markedly reduced. The abundances of the genera *Klebsiella* and *Delftia* in ileum, colon and fecal samples were substantially increased in AMNV-treated mice compared with WT mice (Fig. 6e–g). In contrast, the abundance of the probiotic *Lactobacillus* was significantly decreased in ileum, colon and fecal samples after antibiotic treatment. The abundance of *Enterococcus* was substantially increased in ileum samples from AMNV-treated mice but was not altered in colon or fecal samples (Fig. 6e–g).

Two days after the cessation of antibiotics, we challenged the mice with *Klebsiella pneumoniae* (5 × 10^6^ CFUs) via the intragastric route (Additional file 1: Figure S6a). Mice were sacrificed at 0 h, 24 h, 48 h, and 72 h. Microbiota-depleted mice showed a significant increase in the bacterial burden in the lung at 24 h and 48 h compared with WT mice after challenge with *Klebsiella pneumoniae* (Additional file 1: Figure S6b). The rates of local gut epithelial necrosis and substantial inflammatory cell infiltration were significantly increased in both of the *Klebsiella pneumoniae*-treated groups but were unaffected by broad-spectrum antibiotic pretreatment (Additional file 1: Figure S6c). We reasoned that antibiotic treatments may alter intestinal epithelium paracellular permeability, which facilitates the translocation of *Klebsiella pneumoniae*. To test this hypothesis, we treated the mice with FITC-dextran to determine intestinal permeability. The serum fluorescence intensities in the WT + KP and AMNV + KP groups were higher than those in the WT and AMNV groups. We also observed that the serum fluorescence intensity in the AMNV + KP group was significantly higher than that in the WT + KP group (Additional file 1: Figure S6d, *P* < 0.05). These findings revealed that *Klebsiella* translocated from the intestine to the lung tissue of AMNV-treated mice, likely due to increased intestinal epithelium paracellular permeability.

## Discussion

In the current study, we observed marked shifts in the fecal bacterial composition of healthy controls compared to septic patients and ICU controls. The original predominant bacteria in the gut microbiota were replaced by the opportunistic infectious bacteria *Klebsiella pneumoniae* and *Enterococcus faecium*. In addition, the gut microbiota composition of survival septic patient returned to a relatively normal state after one week of treatment, while that of non-survival patients did not. Some septic patients developed secondary infections, and most of the secondary infection sites were the blood and lung. Among all the secondary infectious bacteria, CRKP, ranking first among the leading causes of nosocomial infection, accounted for 41.2%; this was speculated to be a probable consequence of broad-spectrum antibiotic use. We analyzed fecal samples from patients with secondary systemic CRKP and VRE infections and found that the time points of systemic infection onset all occurred after the time points of corresponding intestinal bacterial predominance. Importantly, genome homology analysis showed that the dominant CRKP or VRE in the intestinal tract was identical to those isolated from the bloodstream and abdomen. Moreover, translocation of CRKP from the intestine to the lungs in gut microbiota-depleted mice verified our findings. The destruction of the gut barrier caused by antibiotic treatment may be a potential mechanism facilitating the enteral-to-systemic translocation of CRKP. Based on these findings, the pathogens causing nosocomial or secondary infection in septic patients probably originated from excessive intestinal colonization, which was probably associated with broad-spectrum antibiotic administration.

Bacterial translocation has been reported in several previous studies [6, 25]. However, some previous studies found that conditions that contributed to bacterial translocation were mainly present in patients undergoing a major operation or with severe injury [26, 27] as well as in patients with cirrhosis or severe pancreatitis [28, 29]. In this study, we showed that the relative abundances of *Klebsiella pneumoniae* and *Enterococcus faecium* in the intestine after a one-week ICU stay might be associated with the antibiotic therapy. Our mouse experiment indicated that antibiotic treatment was related to the integrity of the gut barrier, which enabled opportunistic enteral pathogens to translocate to systemic sites such as the lungs and bloodstream and allowed *Klebsiella pneumoniae* intestinal colonization. Previous work reported that VRE colonization in the small intestine or cecum might represent a predictive factor that can be detected several days earlier than VRE bloodstream colonization [6]. Our data showed similar results for *Klebsiella pneumoniae* and *Enterococcus faecium*, which were the dominant intestinal microbiota that translocated to systemic organs such as the blood and abdomen in septic patients. These findings indicated that predominant *Klebsiella pneumoniae* or *Enterococcus faecium* colonization in the gut could potentially represent a predictive factor for systemic infection in septic patients.

Previous evidence linking gut-originated bacterial translocation, particularly to the bloodstream or pulmonary system, in septic patients was not sufficient. Dickson and his colleagues showed  that gut-lung bacterial translocation and subsequent alterations in the lung microbiome may represent a common mechanism of sepsis and ARDS pathogenesis [30]. However, that study lacked techniques such as paired metagenomic comparisons for analysis of the homology of gut and lung bacteria. PFGE fingerprinting of Xbal-digested DNA of CRKP was performed by Konstantina to compare samples from rectal swabs and blood culture, but the absolute or relative abundance of *Klebsiella pneumoniae* in the total gut microbiota was completely unknown [25]. In the current study, we used genome sequencing to identify the homology between *Klebsiella pneumoniae* and *Enterococcus faecium* isolated from blood, abdominal samples and the gut; this provides more precise evidence of bacterial translocation from the intestine, inducing secondary systemic infection.

The intestinal microbiota composition is sensitive to many factors [31], such as antibiotic administration and other clinical treatments [32]. A previous study by Pammer proved that antibiotic treatment in mice enabled orally administered VRE to rapidly and entirely replace the normal intestine microbiota [6]. Kontopoulou et al.revealed that treatment with anti-anaerobic antibiotics disturbed the stability of the gut microbiota due to the substantial reduction in the abundance of anaerobic bacteria, leading to CRKP colonization [25]. In the current study, most septic patients were treated with broad-spectrum antibiotics, and the Shannon diversity index of the intestinal microbiota was significantly decreased after one week of hospitalization. The gut microbiota in septic patients was different from that in healthy controls according to PCoA, and the relative abundance of *Firmicutes*, which contains many anaerobic bacterial genera, gradually lost its dominant rank. The abundance of *Anaerostipes*, an obligate anaerobic, butyrate-producing bacterial genus, decreased with sepsis progression, indicating that the anaerobic bacteria host defense mechanism that inhibits the colonization of pathogens was destroyed [33].

Despite the novel findings and clinical implications, the current study had several limitations. First, the number of patients recruited was limited and restricted to a single center; therefore, larger samples and multicenter studies will be needed for further validation in the future. Second, *Enterococcus faecium* isolated from the patients was not used for mouse gavage due to dissatisfactory *Enterococcus faecium* proliferation. Third, although metagenome sequencing revealed that *Klebsiella pneumoniae* dominated the gut microbiota and that its genome was identical to that of strains isolated from the bloodstream, a comparison of bacteria collected prior to and after antibiotic treatment would help clarify whether *Klebsiella pneumoniae* colonized the gut prior to infecting the blood.

## Conclusions

We found that the gut microbiota of patients with sepsis tended to be dominated by *Klebsiella* or *Enterococcus* after broad-spectrum antibiotic treatment. Due to the vulnerability of the disrupted intestinal barrier to opportunistic pathogens, *Klebsiella* and *Enterococcus* might translocate from the gut to the bloodstream and lungs, which were associated with systemic infection and increasing the risk of mortality in septic patients. Thus, high-throughput DNA sequencing of the intestinal microbiota might identify patients at high risk of developing nosocomial and secondary infections.

## Supplementary Information

**Additional file 1**. This file contains descriptions and figures of external data.

**Additional file 2**. This file contains the detailed information of all septic patients.

**Additional file 3**. Types and durations of antibiotic therapy of all septic patients.

## Data Availability

The raw 16S rDNA sequencing data were deposited in the National Omics Data Encyclopedia (NODE, https://www.biosino.org/node/index) under accession numbers OEX010715 and OEX010716. The raw metagenomics data were deposited in NODE under accession number OEX010714, and the assembled genome sequences of cultured *Klebsiella pneumoniae* and *Enterococcus faecium* were deposited in NODE under accession numbers OEZ008360-OEZ008366. The remaining data are available from the corresponding author upon request.

## Abbreviations

AMNV: Ampicillin, metronidazole, neomycin, and vancomycin
APACHE: Acute Physiology and Chronic Health Evaluation
CFU: Colony-forming unit
CRKP: Carbapenem-resistant *Klebsiella pneumoniae*
CRP: C-reactive protein
FMT: Fecal microbiota transplantation
IL-1β: Interleukin-1β
IL-2R: Interleukin-2 receptor
IL-6: Interleukin-6
IL-8: Interleukin-8
IL-10: Interleukin-10
MODS: Multiple organ dysfunction syndrome
NE: Neutrophil
OUT: Operational taxonomic unit
PCoA: Principal component analysis
PCT: Procalcitonin
SNP: Single nucleotide polymorphisms
SOFA: Sequential Organ Failure Assessment
TNF-α: Tumor necrosis factor alpha
VRE: Vancomycin-resistant *Enterococcus faecium*
WBC: White blood cell
