# Supplementary material for: The pathogens of secondary infection in septic patients share a similar genotype to those that predominate in the gut
Source: Crit Care. 2022 Mar 24;26:68. doi: 10.1186/s13054-022-03943-z (PMC8944137; doi:10.1186/s13054-022-03943-z)
Supplement: Supplementary file 1 — Additional file 1. This file contains descriptions and figures of external data. [file 13054_2022_3943_MOESM1_ESM.pdf]

Additional file

Additional table 1. Clinical characteristic of septic and non-septic ICU patients

	Septic patients (n=34)	ICU control patients (n=33)	*P* value
Age (years)	58.50 (58.00, 67.25)	69.00 (50.00, 76.00)	0.241
Gender (male, %)	22 (64.71%)	18 (54.54%)	0.275
28-d mortality (%)	10 (29.41%)	1 (3.03%)	0.004
Complications (N, %)			
Hypertension	18 (52.94%)	17 (51.52%)	0.545
CHD	2 (5.88%)	3 (9.09%)	0.682
COPD	2 (5.88%)	3 (9.09%)	0.486
Asthma	1 (2.94%)	0	0.507
T_2DM	14 (41.76%)	10 (30.30%)	0.251
Special treatment (N, %)			
Vasoactive intervention	5 (14.71%)	1 (3.03%)	0.105
Mechanical Ventilation	20 (58.82%)	3 (9.09%)	<0.001
Renal replacement	5 (14.71%)	0	0.029
Infection sites (N, %)			
Lung	20 (58.82%)	-	-
Abdominal	12 (35.39%)	-	-
Bloodstream	1 (2.94%)	-	-
Urinary	2 (588%)	-	-
Infection pathogens (N, %)			
G^+	7 (20.59%)	-	-
G^-	9 (26.47)	-	-
Unknown	18 (52.94%)	-	-

Abbreviations: CHD, coronary heart disease; COPD, chronic obstructive pulmonary disease; T_2DM, type 2 diabetes

Additional Table 2. Clinical information of survival and non-survival septic patients

	Survival patients (n=24)	Non-survival patients (n=10)	*P* value
Age (years)	53.50 (48.00, 69.25)	64.00 (54.75, 68.25)	0.183
Gender (male, %)	15 (62.50%)	7 (70.00%)	0.498
Complications (N, %)			
Hypertension	12 (50.00%)	6 (60.00%)	0.332
CHD	1 (4.17%)	1 (10.00%)	0.508
COPD	2 (8.34%)	0	0.492
Asthma	1 (4.17%)	0	0.706
T_2DM	9 (37.50%)	5 (50.00%)	0.221
Secondary			
Infection sites (N, %)			
Lung	10 (41.67%)	6 (60.00%)	0.275
Abdominal	0	1 (10.00%)	0.294
Bloodstream	5 (20.83%)	6 (60.00%)	0.036
Urinary	8 (33.33%)	2 (20.00%)	0.367

Additional Table 3. Clinical characteristic of non-survival septic patients on different stages.

	Non-survival Day1	Non-survival Day7	*P* value
SOFA score	6.50 (4.00, 8.75)	7.00 (5.75, 12.75)	0.529
APACHE II score	18.2 ± 5.18	19.7 ± 6.86	0.588
WBC (10^9/L)	10.37 (6.53, 17.08)	15.78 (6.89, 20.89)	0.353
CRP (mg/L)	116.35 (71.68, 334.30)	83.25 (35.25, 204.90)	0.247
PCT (ng/ml)	5.19 (0.79, 31.44)	1.32 (0.41, 7.45)	0.393
TNF-α (pg/L)	19.85 (10.33, 33.85)	16.75 (10.95, 34.98)	0.853
IL-1β (pg/L)	5.00 (5.00, 5.40)	5.00 (5.00, 5.25)	0.853
IL-2R (pg/L)	1061.50 (851.75, 4834.00)	1079.50 (742.75, 3714.75)	0.912
IL-6 (pg/L)	134.075 (12.63, 478.25)	16.25 (8.55, 51.25)	0.089
IL-8 (pg/L)	54.50 (30.50, 19825)	64.00 (28.25, 92.75)	0.998
IL-10 (pg/L)	35.55 (7.40, 255.50)	12.35 (6.85, 18.90)	0.165

Additional Table 4. Clinical characteristic of survival septic patients on different stages.

	Survival Day1 (N = 24)	Survival Day7 (N = 24)	*P* value
SOFA score	4.00 (3.00, 6.75)	4.00 (2.25, 5.75)	0.595
APACHE II score	13.00 (7.25, 18.75)	9.00 (6.25, 14.25)	0.008
WBC (10^9/L)	11.30 (6.52, 15.44)	9.90 (5.40, 13.52)	0.571
CRP (mg/L)	100.05 (49.86, 146.78)	19.20 (9.80, 41.90)	< 0.001
PCT (ng/ml)	1.71 (0.40, 20.06)	0.42 (0.20, 1.23)	0.018
TNF-α (pg/L)	13.50 (9.30, 22.25)	13.30 (8.60, 20.00)	0.497
IL-1β (pg/L)	5.00 (5.00, 5.00)	5.00 (5.00, 5.00)	0.691
IL-2R (pg/L)	1532.30 ± 599.11	998.88 ± 430.89	0.001
IL-6 (pg/L)	27.50 (14.50, 61.30)	19.00 (5.38, 44.85)	0.125
IL-8 (pg/L)	40.00 (21.00, 88.00)	32.00 (12.50, 44.75)	0.180
IL-10 (pg/L)	11.40 (6.50, 14.90)	5.60 (5.00, 14.13)	0.043

Additional Table 5. Correlation between gut microbiota and clinical characteristics

Genus	Parameters	SOFA	APACHEII	WBC	CRP	PCT	TNF-α	IL-1β	IL-2R	IL-6	IL-8	IL-10
Enterococcus	R value	-0.018	0.003	-0.024	-0.076	-0.04	-0.038	-0.062	**-0.193**	-0.105	0.017	-0.091
	P value	0.834	0.971	0.786	0.392	0.651	0.702	0.517	0.043	0.274	0.862	0.346
Klebsiella	R value	0.061	**0.191**	**0.175**	0.024	0.113	0.057	0.014	-0.057	0.114	0.01	0.07
	P value	0.484	0.027	0.046	0.789	0.2	0.564	0.882	0.552	0.234	0.919	0.468
Streptococcus	R value	0.071	0.048	-0.009	0.087	-0.092	-0.107	-0.121	-0.116	-0.033	-0.076	-0.04
	P value	0.414	0.58	0.921	0.325	0.295	0.283	0.208	0.226	0.731	0.432	0.681
Bacteroides	R value	-0.051	-0.112	0.099	0.072	0.139	**0.28**	0.11	0.16	**0.226**	0.108	0.063
	P value	0.556	0.197	0.261	0.413	0.113	0.004	0.253	0.096	0.017	0.262	0.516
Gemmiger	R value	**-0.21**	-0.148	-0.013	0.128	0.106	-0.016	0.081	0.029	0.064	0.127	0.058
	P value	0.015	0.087	0.885	0.148	0.228	0.873	0.403	0.764	0.509	0.186	0.547
Bifidobacterium	R value	-0.005	0.07	-0.034	-0.025	0.064	-0.106	-0.086	0.029	-0.006	-0.008	0.076
	P value	0.954	0.42	0.702	0.78	0.466	0.285	0.373	0.767	0.951	0.937	0.433
Faecalibacterium	R value	**-0.237**	**-0.19**	0.006	0.134	0.049	-0.016	0.161	-0.035	-0.019	0.072	-0.022
	P value	0.006	0.028	0.947	0.129	0.579	0.874	0.092	0.716	0.844	0.456	0.822
Erysipelatoclostridium	R value	**-0.171**	-0.107	-0.065	-0.041	-0.036	-0.004	-0.065	-0.053	0.01	0.05	-0.118
	P value	0.048	0.22	0.464	0.647	0.679	0.965	0.502	0.583	0.92	0.605	0.218
Parabacteroides	R value	-0.137	-0.114	0.052	0.103	0.098	0.108	0.012	0.075	0.163	0.126	0.114
	P value	0.113	0.189	0.559	0.244	0.265	0.276	0.899	0.436	0.088	0.189	0.237
Blautia	R value	-0.053	-0.069	-0.013	0.159	0.075	0.045	0.046	0.07	0.113	**0.224**	0.154
	P value	0.545	0.43	0.883	0.071	0.394	0.65	0.633	0.468	0.241	0.019	0.109
Mediterraneibacter	R value	-0.108	-0.158	-0.077	0.073	0.057	-0.097	0.019	-0.077	0.011	0.045	0.008
	P value	0.215	0.069	0.379	0.41	0.517	0.329	0.846	0.423	0.91	0.642	0.936
Ruthenibacterium	R value	0	0.041	-0.059	0.098	0.118	0.027	0.05	0.081	0.093	**0.246**	0.097
	P value	0.999	0.636	0.501	0.269	0.179	0.79	0.604	0.401	0.336	0.01	0.312
Pseudomonas	R value	-0.034	0.054	0.071	-0.038	-0.041	0.078	-0.016	-0.043	-0.064	-0.177	-0.146
	P value	0.692	0.537	0.42	0.668	0.644	0.435	0.87	0.655	0.509	0.064	0.128
Enterocloster	R value	-0.024	-0.023	0.002	**0.208**	0.109	-0.01	0.089	0.098	0.103	**0.256**	0.114
	P value	0.782	0.789	0.978	0.018	0.217	0.918	0.355	0.31	0.285	0.007	0.237
Agathobacter	R value	0.098	0.05	-0.04	0.012	0.098	0.018	0.135	0.027	0.041	-0.037	0.09
	P value	0.261	0.565	0.653	0.89	0.265	0.86	0.16	0.783	0.672	0.704	0.349
Lactobacillus	R value	0.061	0.033	0.026	-0.008	-0.051	-0.061	-0.166	-0.136	-0.018	**-0.237**	-0.089
	P value	0.485	0.705	0.767	0.931	0.56	0.544	0.083	0.158	0.853	0.013	0.356
Rothia	R value	**-0.189**	**-0.214**	-0.114	-0.02	**-0.239**	-0.088	-0.021	-0.185	-0.095	-0.071	-0.149
	P value	0.029	0.013	0.196	0.822	0.006	0.375	0.83	0.053	0.321	0.458	0.119
Alistipes	R value	-0.097	-0.013	-0.003	0.128	0.071	0.102	-0.005	0.122	0.178	**0.197**	0.14
	P value	0.266	0.885	0.974	0.145	0.423	0.307	0.958	0.205	0.062	0.039	0.146
Ruminococcus	R value	0.085	0.126	0.012	**0.192**	**0.217**	0.13	0.029	0.122	0.097	**0.189**	**0.219**
	P value	0.33	0.148	0.891	0.029	0.013	0.192	0.761	0.204	0.315	0.048	0.022
Agathobaculum	R value	-0.087	-0.12	0.111	0.035	0.083	-0.044	0.111	-0.011	-0.086	0.142	-0.036
	P value	0.317	0.168	0.208	0.695	0.345	0.66	0.25	0.911	0.371	0.138	0.709
Coprococcus	R value	-0.078	-0.096	0.036	**0.177**	0.087	-0.04	0.156	-0.066	0.062	0.066	0.086
	P value	0.372	0.269	0.684	0.044	0.321	0.685	0.104	0.494	0.521	0.496	0.372
Veillonella	R value	0.031	-0.111	0.049	0.037	-0.117	-0.06	-0.009	-0.137	0.028	0.005	-0.025
	P value	0.72	0.201	0.575	0.679	0.182	0.544	0.929	0.153	0.773	0.962	0.796
Anaerostipes	R value	0.012	0.043	-0.01	0.103	0.119	0.015	0.019	0.149	-0.012	0.113	0.037
	P value	0.887	0.619	0.907	0.244	0.174	0.877	0.846	0.121	0.898	0.238	0.701
Collinsella	R value	-0.024	0.028	0.051	0.164	0.159	-0.058	-0.012	-0.054	**0.209**	-0.029	0.09
	P value	0.78	0.75	0.56	0.063	0.07	0.56	0.903	0.572	0.028	0.764	0.348
Weissella	R value	0.013	0.076	0.03	-0.021	-0.074	0	**-0.209**	-0.085	-0.061	**-0.268**	-0.083
	P value	0.885	0.385	0.734	0.81	0.398	0.998	0.028	0.379	0.527	0.005	0.391
Clostridium sensu stricto	R value	0.084	0.131	0.051	0.021	0.166	0.006	0.06	0.024	-0.003	-0.019	0.118
	P value	0.335	0.132	0.563	0.814	0.058	0.954	0.53	0.806	0.971	0.842	0.219
Staphylococcus	R value	**0.189**	0.149	0.044	0.038	-0.028	0.09	-0.061	-0.034	0.002	0.114	-0.024
	P value	0.029	0.086	0.616	0.671	0.75	0.365	0.524	0.724	0.981	0.235	0.8
Dorea	R value	0	0.038	0.069	**0.181**	**0.231**	0.063	-0.105	0.091	0.098	-0.028	0.101
	P value	1	0.666	0.431	0.04	0.008	0.53	0.275	0.346	0.309	0.773	0.293
Eggerthella	R value	0.053	0.026	-0.023	**0.191**	0.12	0.014	-0.038	0.009	0.051	0.13	0.012
	P value	0.544	0.765	0.791	0.029	0.171	0.892	0.692	0.926	0.597	0.177	0.899
Schaalia	R value	-0.121	-0.15	-0.011	0.113	-0.098	-0.082	-0.051	-0.162	-0.036	-0.076	-0.133
	P value	0.164	0.084	0.905	0.199	0.266	0.408	0.595	0.091	0.711	0.43	0.166

Additional Table 6. The multivariate linear regressions of gut microbiota and septic/non-septic patients at Day7 adjusted for SOFA and APACHE II scores .

Group	Shannon		Simpson		Chao		ACE	
	β (95% CI)	*P*	β (95% CI)	*P*	β (95% CI)	*P*	β (95% CI)	*P*
Septic Patients or not (Ref: No)	-0.637 (-1.136, -0.137)	0.013	0.504 (0.145, 0.864)	0.007	-0.311 (-0.792, 0.169)	0.2	-0.310 (-0.790, 0.170)	0.202
SOFA	0.034 (-0.07, 0.137)	0.518	-0.005 (-0.080, 0.069)	0.884	0.054 (-0.045, 0.154)	0.28	0.054 (-0.046, 0.154)	0.282
APACHE II	-0.021 (-0.073, 0.03)	0.414	0.011 (-0.026, 0.048)	0.552	-0.015 (-0.065, 0.035)	0.545	-0.015 (-0.064, 0.035)	0.558

Figures

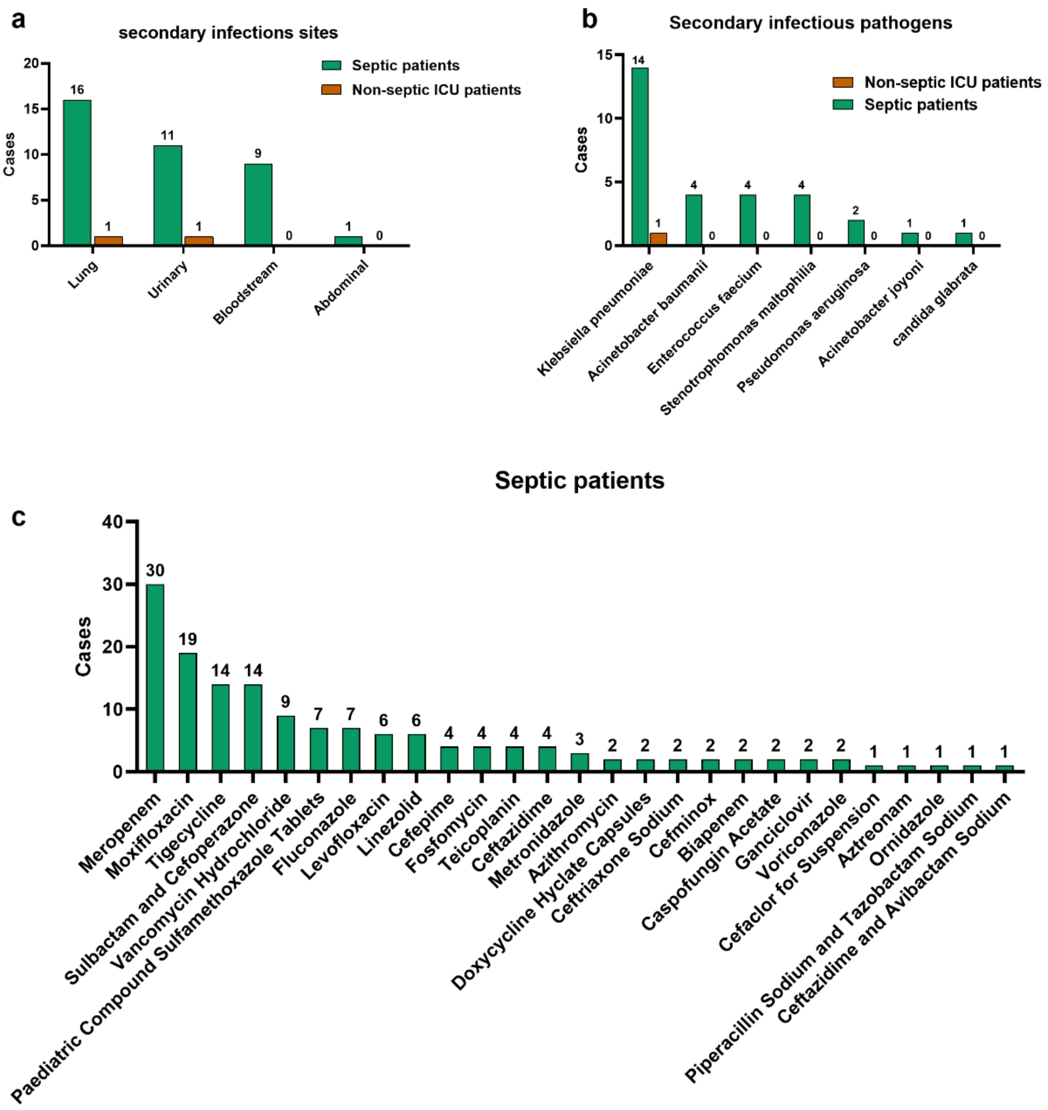

Additional Figure 1. Secondary infection sites **(a)**, pathogens **(b)** and cases of antibiotic application **(c)** in septic and non-septic ICU patients.

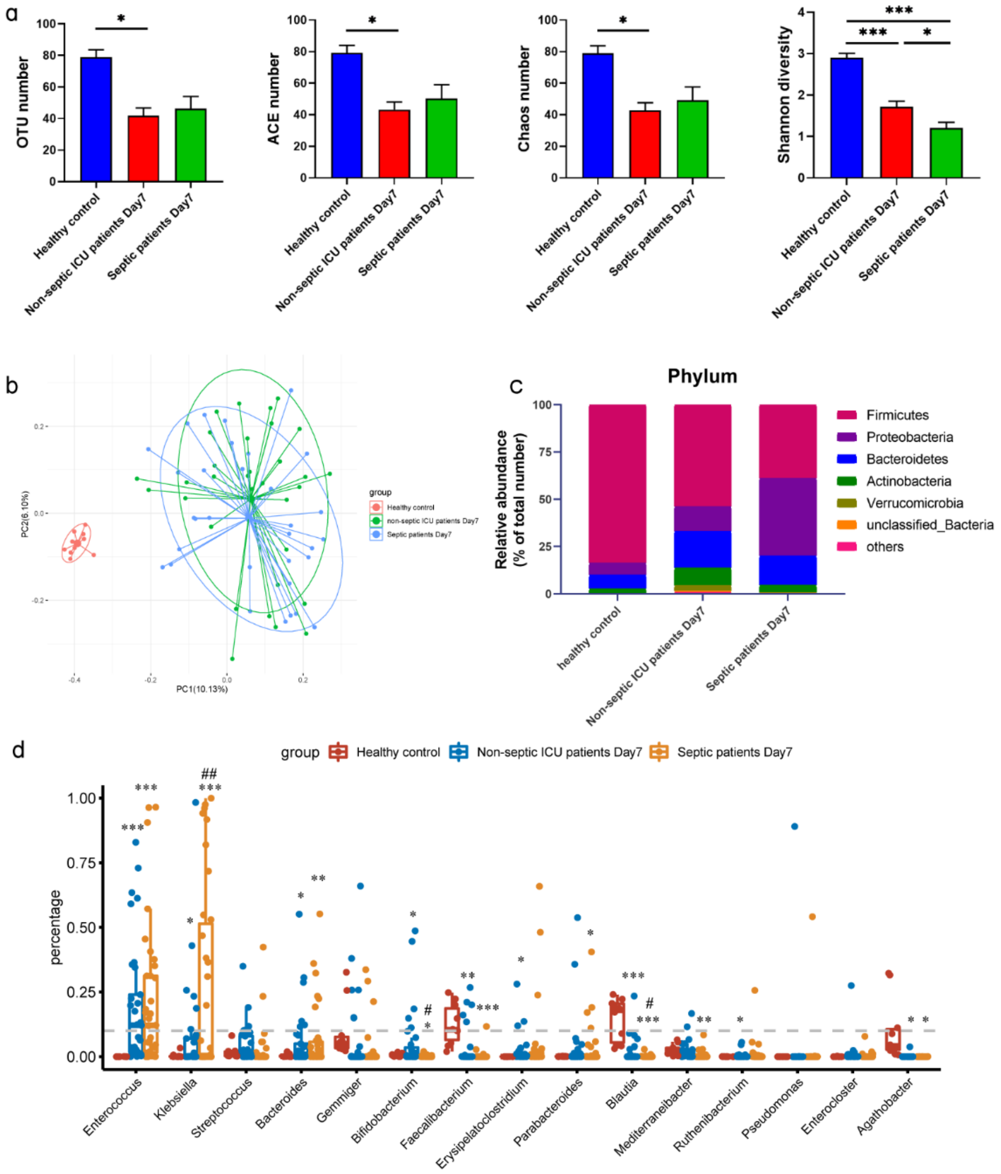

Additional Figure 2. **a.** Alpha diversity analysis based on OTU, ACE and Chao numbers and beta diversity analysis based on the Shannon diversity index among the three groups. **b.** Principal coordinate analysis (PCoA) using unweighted UniFrac distances showed significant differences in the microbiota composition of the three groups. **c**. Mean proportions of phylum compositions in the three groups. d. Proportion of genus composition of the three groups. *indicates comparisons with healthy controls, # indicates comparisons with non-septic ICU patients on Day 7. *, $P < 0.05$, **, $P < 0.01$, ***, $P < 0.001$, and #, $P < 0.05$.

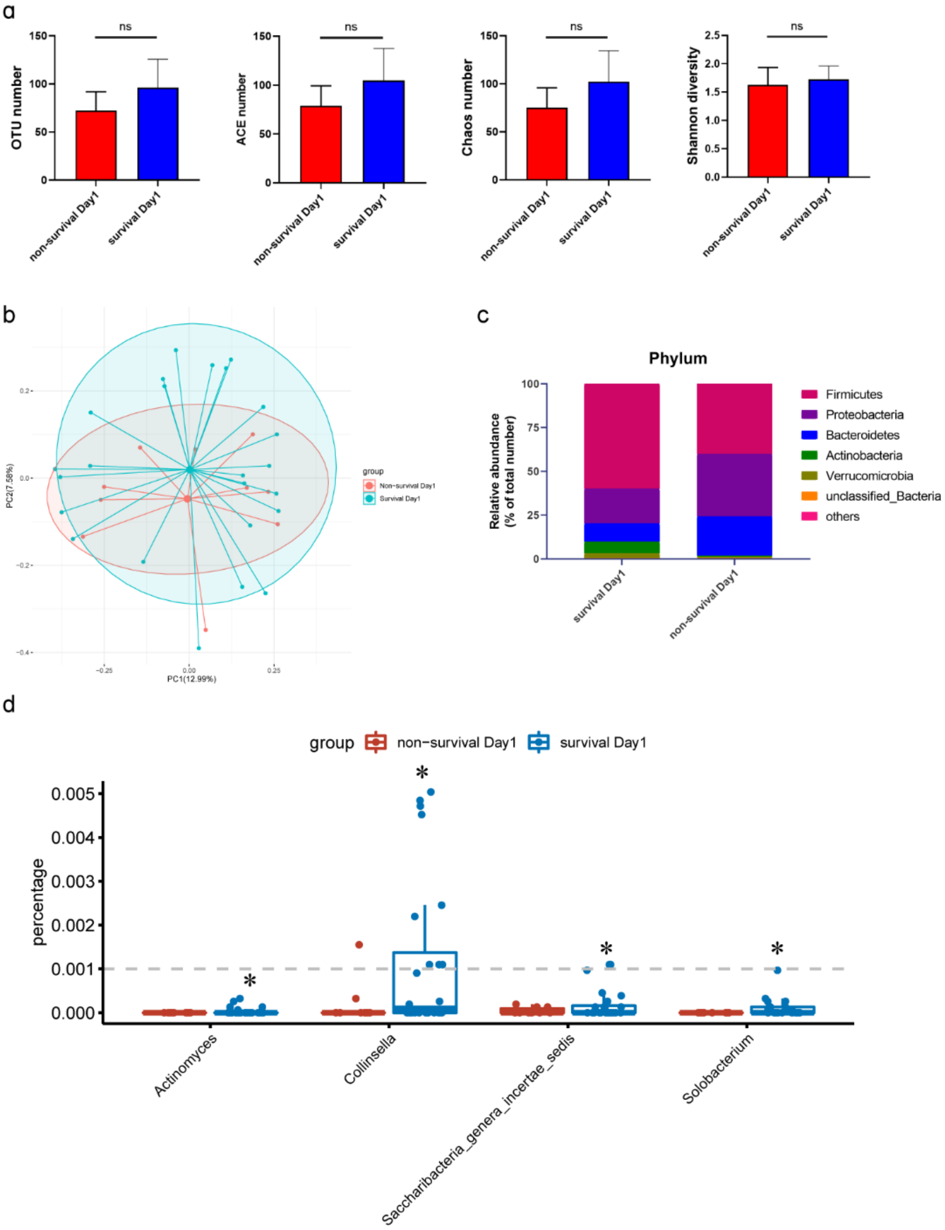

Additional Figure 3. Gut microbiota alterations between survival and non-survival septic patients on admission day.

A. Alpha diversity analysis based on OTU, ACE and Chao numbers and beta diversity analysis based on the Shannon diversity index between survival and non-survival septic patients on the day 1. **B.** Principal coordinate analysis (PCoA) using unweighted UniFrac distances showed significant differences in the microbiota composition of the groups. **C**. Mean proportions of phylum compositions in the two groups. **D**. Proportion of genus composition of the two groups. *, $P < 0.05$, **, $P < 0.01$, ***, $P < 0.001$, $^{\#}$, $P < 0.05$.

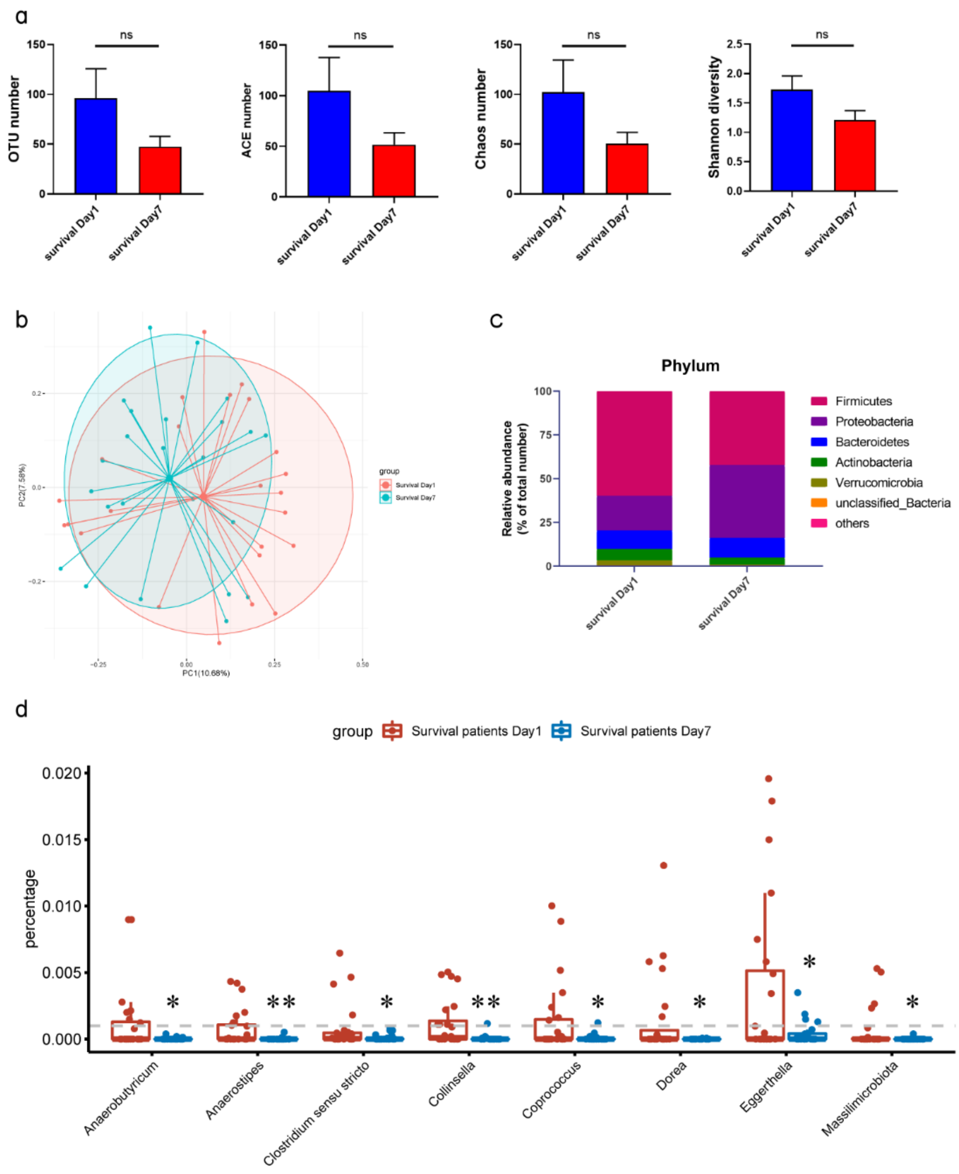

Additional Figure 4. Gut microbiota alterations of survival septic patients on different stages

A. Alpha diversity analysis based on OTU, ACE and Chaos number and Beta diversity analysis based on Shannon diversity between survival septic patients on the day 1 and day 7. **B.** Principal coordinate analysis (PCoA) using unweighted UniFrac distances showed significant differences in the microbiota composition of the groups. **C**. Mean proportions of phylum compositions in the two groups. **D**. Proportion of genus composition of the two groups. *, $P < 0.05$, **, $P < 0.01$, ***, $P < 0.001$, $^{\#}$, $P < 0.05$.

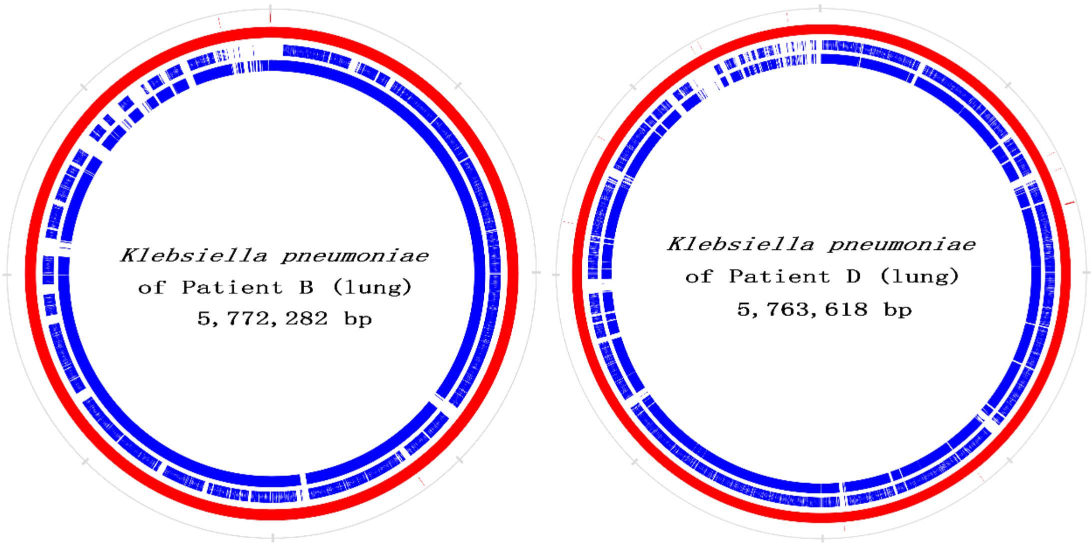

Additional Figure 5. Comparison of the intestinal metagenomic sequences and cultured bacteria genome from the lung of Patient B and D. Using the concatenated draft genome of cultured bacteria from each patient as a reference, the intestinal metagenomic sequences were mapped to the reference, and SNPs are illustrated in the first (outer) circle. The second circle represents the region of the reference genome covered by the metagenome sequences (red). The third and fourth circles (innermost) show the genome comparison result for one randomly downloaded complete genome from NCBI with the reference draft genome (blue), with the third circle representing SNPs and the fourth circle representing coverage. The gaps in the circles indicate that this region was not covered by a metagenomic sequence (the second circle) or other known genomes (the fourth circle).

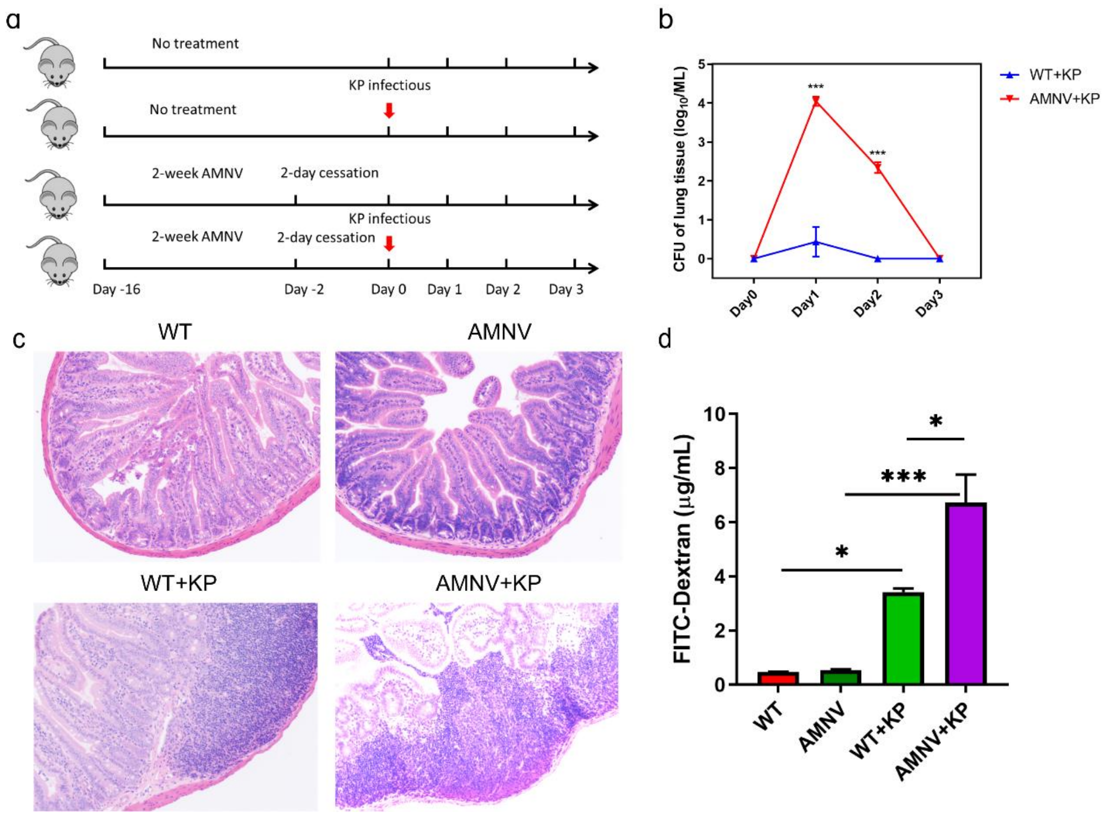

Additional Figure 6. Protective role of the gut microbiota against Klebsiella pneumonia colonization

A. Experimental design. Group of six wild-type mice were treated for 2 weeks with broad-spectrum antibiotics (ampicillin, neomycin, metronidazole and vancomycin) in drinking water compared with untreated controls. Two days after cessation, mice received an oral administration challenge with 5×10^6 colony forming units (CFU) of CRKP. **B**. CFU of lung tissue after CRKP oral administration in AMNV and WT mice. **C**. H&E staining after CRKP pulmonary infection between AMNV and WT mice. **D**. Intestinal permeability assessment by detecting serum FITC-Dextran level. *, $P < 0.05$, **, $P < 0.01$, ***, $P < 0.001$.
